# Plasma-Driven Single-Atom Catalysis: From Synthesis to Catalytic Reactions

**DOI:** 10.1021/acs.nanolett.6c00821

**Published:** 2026-04-02

**Authors:** Haifeng Qi, Weitao Wang, Xin Tu

**Affiliations:** † Department of Electrical Engineering and Electronics, 4591 University of Liverpool, Liverpool L69 3GJ, U.K.

**Keywords:** Plasma catalysis, Single-atom catalyst, Plasma-assisted synthesis, Surface engineering, Fuel and chemical synthesis

## Abstract

The integration of nonthermal plasma (NTP) with single-atom catalysis has recently emerged as a highly promising yet largely unexplored frontier in heterogeneous catalysis. NTP generates highly nonequilibrium reaction environments rich in energetic electrons, radicals, and excited species, while single-atom catalysts (SACs) provide atomically precise active sites with tunable electronic structures and maximized metal utilization. The convergence of these two fields enables unconventional reaction pathways and catalytic behaviors that are not readily accessible under conventional thermal conditions. However, research in this area remains at an early stage, and a systematic understanding of plasma-driven single-atom catalysis (PSAC) is still lacking. In this Review, we provide a comprehensive overview of PSAC, with a particular focus on both plasma-assisted synthesis of SACs and plasma-driven catalytic reactions over isolated metal sites. We summarize recent advances in plasma-enabled atom dispersion, defect engineering, and stabilization strategies and discuss how plasma excitation fundamentally alters reaction mechanisms. Through critical analysis of current achievements and remaining challenges, this Review highlights key opportunities for future research and provides a conceptual framework for the rational design of PSAC systems. We anticipate that the insights presented herein will stimulate further exploration of PSAC synergy and accelerate the development of next-generation catalytic technologies for sustainable fuel and chemical production.

## Graphical abstract

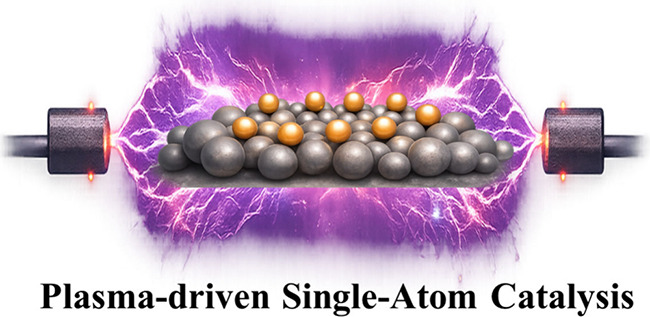

## 1 Fundamentals and Emerging Concepts of Plasma-Driven Single-Atom Catalysis

Single-atom catalysts (SACs), a concept first proposed by Zhang, Li, Liu, and co-workers in 2011,^1^ have emerged as a frontier in heterogeneous catalysis due to their high metal utilization, well-defined coordination environments, and tunable electronic structures.^2–16^ These characteristics bridge the gap between homogeneous, enzyme, and heterogeneous catalysis, enabling unprecedented control over catalytic activity and selectivity.^17–27^ However, the synthesis of SACs with high loading, precise coordination, and long-term stability remains challenging, largely constrained by thermodynamic limitations and kinetic aggregation during conventional thermal or wet-chemical processes.^28–33^ Therefore, developing alternative methods that allow kinetic trapping (i.e., rapid immobilization of metal atoms on supports before thermodynamically driven aggregation), dynamic stabilization, and controlled reconstruction of single-atom sites is critical for the rational design of SACs.

Plasma, often referred to as the fourth state of matter, contains a mixture of energetic electrons, ions, radicals, and electronically or vibrationally excited species. In particular, nonthermal plasma (NTP) has attracted increasing interest as an emerging solution for both catalyst synthesis and chemical reactions.^34–37^ A key advantage of NTP lies in its pronounced nonequilibrium character, with electron temperatures far exceeding those of ions and neutral species.^38–43^ This nonequilibrium chemistry enables highly selective bond activation without bulk heating of the system. Unlike thermal activation, NTP provides access to alternative reaction pathways under nonequilibrium conditions, enabling bond activation, surface modification, and atomic rearrangement at low temperatures and ambient pressure.^44–49^

The physicochemical behavior of plasma-catalytic systems is governed by several key plasma parameters, including electron temperature, electron density, reduced electric field strength, and the distribution of reactive species. Among these, electron temperature governs electron-impact reactions such as excitation, dissociation, and ionization of molecules, while the reduced electric field determines the electron energy distribution function, thereby controlling the dominant reaction pathways. A range of diagnostic techniques have been developed to probe plasma properties, including optical emission spectroscopy, Langmuir probes, and laser-based diagnostics such as laser-induced fluorescence. These approaches provide critical insights into the generation of reactive plasma species and their interactions with catalytic surfaces. Within plasma-catalytic systems, NTP can (i) generate reactive species in the gas phase, (ii) modify catalyst surfaces through ion bombardment or radical adsorption, and (iii) induce electronic excitation and transient electric fields at catalyst interfaces.^50^ Collectively, these effects relax thermodynamic constraints and establish kinetic regimes unattainable in conventional catalysis.

The convergence of plasma science and single-atom catalysis has given rise to a new paradigm, termed plasma-driven single-atom catalysis (PSAC), in which plasma serves both as an efficient tool for catalyst synthesis and as an active driver of catalytic reactions. Within this framework, plasma endows SACs with capabilities that are difficult or even unattainable using conventional approaches. On the synthesis side, plasma enables nonequilibrium coordination control, allowing low-temperature atom trapping that suppresses sintering, access to low-coordinated atomic configurations, and the generation of defects and anchoring sites that strengthen metal–support interactions and stabilize isolated atomic sites. During plasma-assisted reactions, SACs act as highly selective centers that interact synergistically with plasma-generated reactive species. Isolated atomic sites can selectively activate radicals, stabilize key intermediates, and steer reaction pathways, while plasma continuously regenerates reactive species under mild conditions. This cooperative interplay enables enhanced activity, improved selectivity and access to unconventional reaction pathways in challenging transformations involving inert small molecules (e.g., N_2_ and CH_4_), and the activation of high-carbon-chain plastics.

While plasma catalysis and single-atom catalysis have been extensively reviewed individually, a comprehensive examination of their integration as PSAC is still lacking. This Review addresses this gap by summarizing recent advances in PSAC, with a particular focus on plasma-assisted synthesis strategies, plasma-driven catalytic reactions, and emerging perspectives for future development (Figure 1).

**Figure 1:**
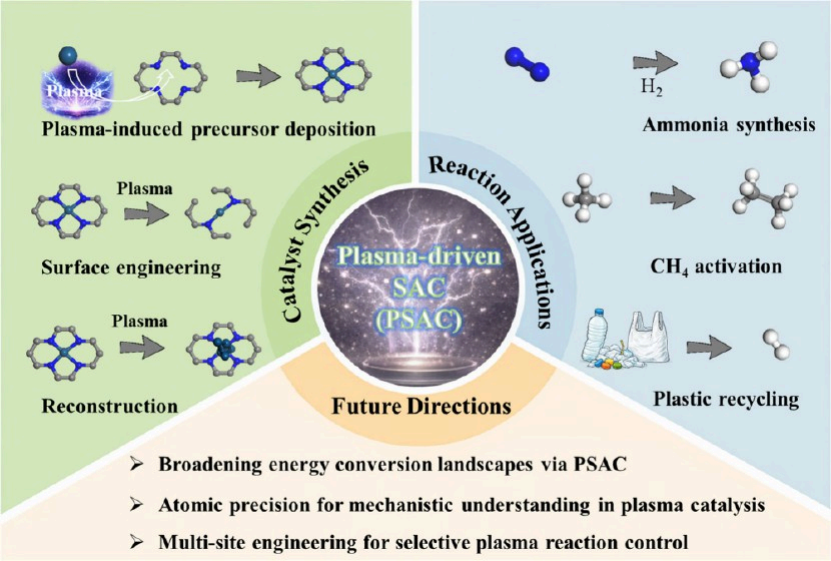
Overview of plasma-assisted synthesis strategies, plasma-driven catalytic reactions, and emerging perspectives for future development.

## 2 Plasma-Assisted Synthesis of SACs

NTP offers unique advantages for the synthesis of SACs by generating highly reactive species, including energetic electrons, radicals, ions, and electronically or vibrationally excited molecules, which enable atomically precise metal manipulation (Figure 2). These plasma-generated species interact with both metal precursors and catalyst supports, creating a highly dynamic and nonequilibrium chemical environment. Energetic electrons can induce rapid precursor dissociation and reduction, while ion bombardment and radical adsorption locally modify surface coordination structures and defect densities. Such interactions also facilitate charge transfer between the support and metal atoms, tuning the oxidation state and electronic structure of isolated metal centers and strengthening metal–support interactions. As a result, plasma exposure can dynamically regulate both the electronic configuration and the coordination environment of single atoms.

**Figure 2:**
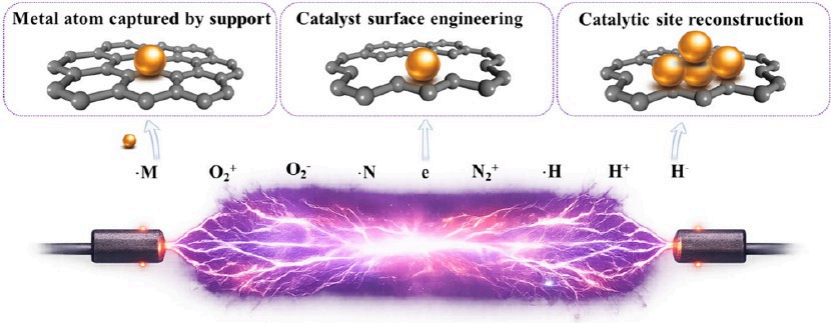
Mechanistic schematic of plasma-assisted dynamic evolution of SACs.

Under these conditions, plasma-generated species facilitate several key processes: (i) trapping of metal atoms on supports through strong anchoring; (ii) surface engineering to tailor coordination environments; (iii) controlled reconstruction of metal sites, including the migration of isolated atoms into clusters or vice versa (Figure 2). Overall, NTP provides a rapid, nonequilibrium pathway for controllable and stable single-atom dispersion that is difficult to achieve using conventional thermal methods.

### 2.1 Plasma-Induced Precursor Deposition

#### 2.1.1 Synthesis of High-Loading SACs

Plasma-induced precursor deposition provides a powerful route for the synthesis of high-loading SACs, enabling efficient metal incorporation while effectively suppressing aggregation. Conventional SACs generally have metal loadings below ∼1 wt %, while high-loading SACs maintain atomic dispersion at ≥1 wt % metal. Under nonequilibrium plasma conditions, metal-containing precursors are rapidly activated and fragmented by energetic electrons and reactive radicals, allowing metal species to be kinetically trapped as isolated atoms on the support surface. This plasma-enabled process decouples metal loading from thermodynamically driven nucleation, facilitating uniform atomic dispersion even at elevated metal contents. Moreover, the low-temperature nature of plasma deposition preserves the structural integrity of supports and provides precise control over both metal loading and spatial distribution, offering a distinct advantage over conventional thermal deposition methods for constructing high-density single-atom catalytic sites.

Rao et al. developed a plasma bombing strategy for the synthesis of high-loading SACs, offering a nonequilibrium alternative to conventional wet-chemical and thermal methods.^51^ In this process, metal salts are excited and stripped into mobile single-metal atoms under intense plasma bombardment, which are simultaneously trapped and stabilized by defect-rich, nitrogen-containing sites on carbon supports (Figure 3a). This unique coupling of metal atom generation under plasma conditions enables rapid trapping and stabilization of isolated metal atoms, effectively suppressing aggregation even at high metal loadings (e.g., Fe up to 8.5 wt %). Notably, this strategy is both general and scalable, and has been successfully extended to multiple transition metals, including Fe, Mn, and Ni. As a representative example, the plasma-synthesized Fe/NC catalyst exhibits exceptional oxygen reduction reaction (ORR) activity and durability, outperforming commercial Pt/C in both rotating disk electrode and zinc–air battery tests. Combined spectroscopic analyses and density functional theory (DFT) calculations identify pyridinic Fe–N_4_ moieties as the dominant active sites and reveal an adsorbate-induced spin-crossover mechanism governing ORR kinetics.

**Figure 3:**
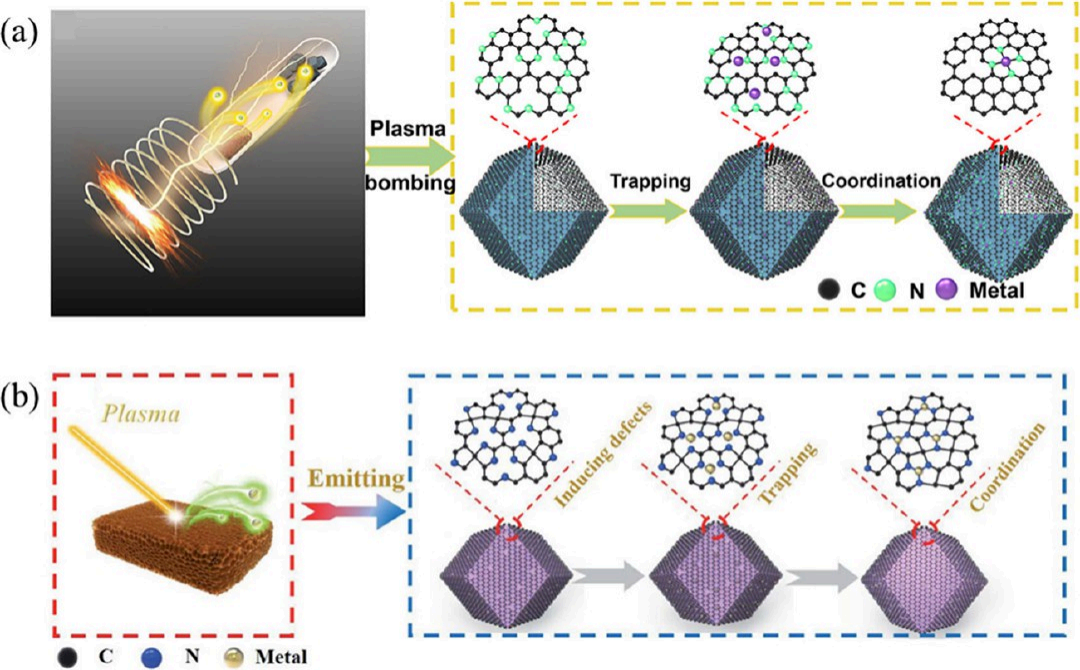
Plasma-enabled strategies for the synthesis of high-loading SACs. (a) Plasma bombardment strategy, where energetic plasma species bombard metal nanoparticles, inducing atom dispersion and anchoring of isolated metal atoms on the support. Reproduced with permission from ref 51. Copyright 2022 Elsevier. (b) Plasma-etching strategy, in which plasma treatment selectively removes surface atoms and generates defect sites that stabilize high-density single-atom metal species. Reproduced with permission from ref 52. Copyright 2022 Royal Society of Chemistry.

Similarly, plasma-etching approaches employ nitrogen plasma to simultaneously etch bulk metal precursors (e.g., Cu foam) into mobile single-metal atoms and induce abundant defects and nitrogen functionalities on carbon supports, enabling in situ trapping and stabilization of isolated metal atoms via strong metal–nitrogen coordination (Figure 3b).^52^ As a representative example, the resulting Cu-SAC/NC exhibits uniformly dispersed Cu single atoms with well-defined Cu–N_3_ coordination environment, achieving outstanding ORR activity and durability, outperforming commercial Pt/C in both rotating disk electrode tests and zinc–air battery devices. Importantly, this scalable and tunable plasma-etching strategy enables gram-scale synthesis of various single- and dual-metal SACs by simply varying metal precursors and plasma parameters, demonstrating the versatility of nonequilibrium plasma as an emerging solution for high-performance SAC fabrication.

Overall, plasma bombing, arc plasma deposition and plasma-etching exemplify complementary nonequilibrium strategies for constructing SACs. In these approaches, plasma enables high-loading atomic dispersion through rapid precursor activation and defect-mediated trapping. Collectively, these advances establish plasma as a powerful and versatile platform for the scalable synthesis and rational design of SACs, overcoming the thermodynamic and kinetic limitations of conventional synthesis methods.

#### 2.1.2 Versatile and Ultrafast Synthesis of Atom-Defined Catalysts via Plasma Treatment

The synthesis of atom-defined SACs with controlled coordination environments remains challenging for conventional thermal and wet-chemical methods, which are often time-consuming and constrained by thermodynamic equilibrium. Plasma treatment provides an ultrafast and nonequilibrium alternative, enabling rapid atomic activation and coordination regulation under mild conditions. Such plasma-enabled synthesis routes provide access to metastable atomic configurations and accelerate the rational design of high-performance SACs beyond conventional synthetic limitations.

A representative example is the continuous-flow solution plasma (CSP) strategy, which enables atom-economic and scalable synthesis of both single-atom and dual-atom catalysts, offering a sustainable alternative to conventional reduction-based routes.^53^ In this process, solution plasma generates high-flux hydrated electrons and reactive hydrogen species that rapidly reduce metal precursors within an extremely short residence time (∼0.03 s). Simultaneously, plasma exposure induces abundant anchoring sites on CeO_2_ supports, allowing immediate trapping and stabilization of isolated metal atoms (Figure 4a). This coupled reduction-anchoring mechanism achieves exceptional metal utilization efficiencies (>97%) and effectively suppresses metal aggregation, even in multimetallic systems that are difficult to control using traditional approaches. Benefiting from its continuous-flow configuration and nonequilibrium nature, the CSP strategy exhibits high versatility and broad applicability, enabling the synthesis of a wide range of noble-metal SACs (Au, Rh, Pd, Ru, and Pt) as well as precisely programmed dual-atom catalysts such as Au_1_Rh_1_/CeO_2_ and Au_1_Pd_1_/CeO_2_. Among these, Au_1_Rh_1_/CeO_2_ exhibits remarkable low-temperature water–gas shift activity, achieving 50% CO conversion at room temperature under light irradiation, which is attributed to synergistic dual-atom interactions that promote lattice oxygen activation and regulate intermediate adsorption.

**Figure 4:**
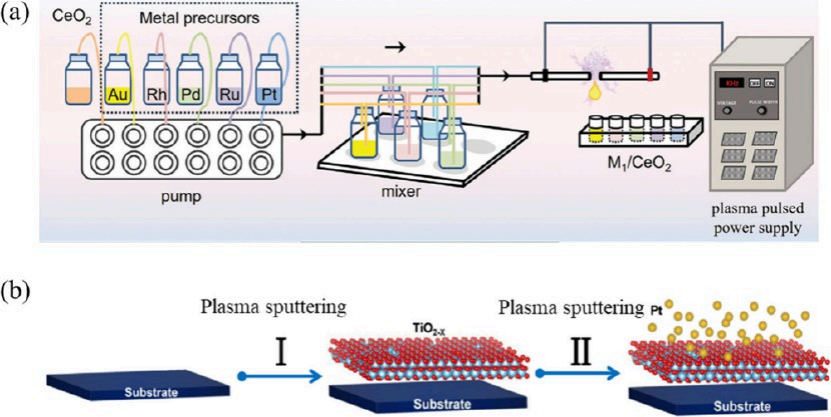
Versatile plasma-assisted strategies for the synthesis of atom-defined catalysts. (a) CSP strategy, in which liquid-phase plasma enables rapid nucleation and dispersion of metal species, yielding atomically dispersed catalysts. Reproduced with permission from ref 53. Copyright 2024 Wiley. (b) Plasma sputtering approach, where metal atoms are directly generated and deposited onto catalyst supports, enabling precise control over atomic dispersion and catalyst structure. Reproduced with permission from ref 54. Copyright 2021 Elsevier.

Beyond solution-phase plasma routes, solid-phase plasma techniques further expand the toolbox for ultrafast SAC synthesis. Tian et al. demonstrated a plasma sputtering strategy for the rapid synthesis of Pt SACs with high intrinsic activity toward the hydrogen evolution reaction (HER), highlighting the unique advantages of nonequilibrium plasma in noble-metal utilization.^54^ In this work, Pt single atoms are deposited directly onto TiO_2–*x*_ supports through alternating plasma sputtering of Pt and support targets at ambient temperature (Figure 4b), enabling catalyst fabrication within minutes or even seconds without chemical reagents. The plasma sputtering process generates highly energetic Pt species that are kinetically trapped as isolated atoms through strong Pt–O coordination on TiO_2–*x*_, effectively suppressing nanoparticle formation even at practical loadings (∼1.69 wt %). Structural characterizations confirm the exclusive presence of atomically dispersed, predominantly oxidized Pt species. DFT calculations further identify Pt–O sites on the rutile TiO_2_ (010) surface as the true HER active centers with near-thermoneutral hydrogen adsorption. Benefiting from full atomic utilization, the resulting Pt/TiO_2–*x*_ catalyst exhibits outstanding HER performance in acidic media, with a turnover frequency of ∼39.6 s^–1^ and mass activity exceeding that of commercial Pt/C catalysts, together with superior durability. This study establishes plasma sputtering as a rapid, energy-efficient, and scalable platform for constructing high-performance single-atom electrocatalysts beyond the limitations of conventional synthesis routes.

### 2.2 Plasma-Assisted Surface Engineering of SACs

Plasma-assisted surface engineering provides a promising solution for tailoring the physicochemical properties of catalyst supports to stabilize single-atom species. Owing to its nonequilibrium nature, plasma treatment can introduce abundant surface defects, heteroatom dopants, and functional groups that serve as robust anchoring sites for isolated metal atoms. In addition, plasma exposure can tailor surface electronic structures and surface wettability, thereby strengthening metal–support interactions and suppressing atom migration and aggregation. Such plasma-enabled surface engineering strategies are crucial for constructing high-performance SACs.

#### 2.2.1 Plasma-Assisted Coordination Engineering of Single Atoms

Plasma enables direct regulation of the local coordination environment of single-atom sites under nonequilibrium conditions, allowing access to metastable configurations that are difficult to achieve via conventional thermal treatment. By selectively tuning the local atomic surroundings, plasma can optimize the electronic structure, adsorption properties, and catalytic activity of SACs, thereby enhancing reaction kinetics while suppressing undesired side reactions.

Jia et al. reported a plasma-assisted nitrogen-vacancy-induced coordinative reconstruction strategy to tailor the local coordination environment of single-atom Ni catalysts for efficient electrochemical CO_2_ reduction.^55^ Using microwave-induced plasma treatment, nitrogen atoms surrounding preformed Ni–N–C sites are selectively removed under nonequilibrium conditions, generating abundant nitrogen vacancies and driving structural reconstruction from pyrrolic N-coordinated Ni species to a more active, unsaturated pyridinic Ni–N_2_ configuration (Figure 5a). This plasma-enabled defect engineering simultaneously preserves atomic dispersion while precisely lowering the coordination number of Ni, creating highly unsaturated and electronically activated sites. The resulting defective Ni–N_2_ sites exhibit significantly enhanced CO_2_ adsorption and activation capability, achieving a high CO Faradaic efficiency of 96% at a mild overpotential, along with markedly increased partial current density compared with their N-saturated counterparts. DFT calculations reveal that the nitrogen-vacancy-induced unsaturated coordination environment reduces the energy barrier for *COOH formation and facilitates dynamic displacement of Ni atoms during catalysis, accelerating reaction kinetics while suppressing competing hydrogen evolution.

**Figure 5:**
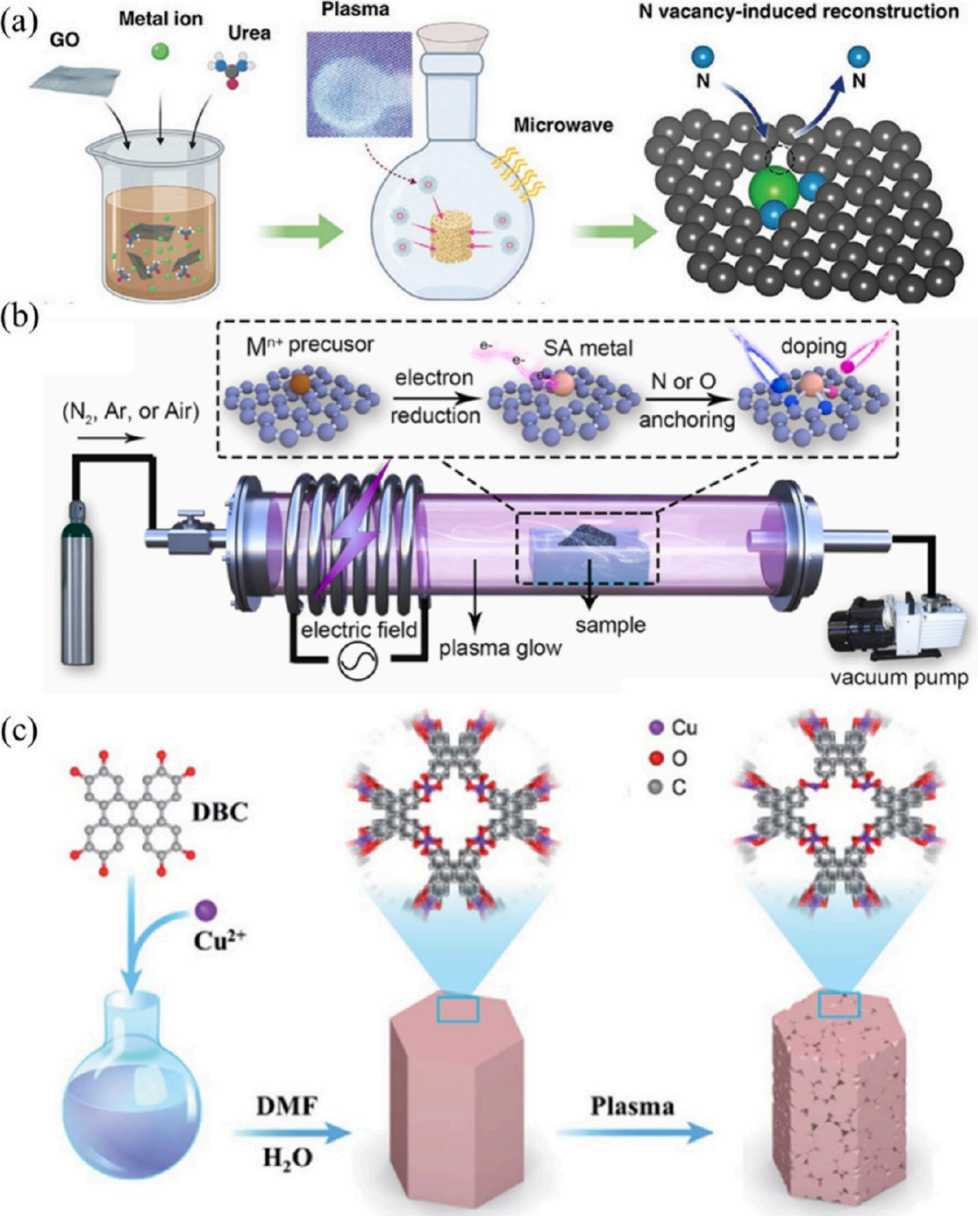
Plasma-assisted coordination engineering of SACs. (a) Nitrogen-vacancy-induced coordinative reconstruction of Ni SACs, where plasma treatment generates nitrogen vacancies that modify the coordination environment of Ni sites. Reproduced with permission from ref 55. Copyright 2021 Wiley. (b) NTP-enabled synthesis of supported Pt SACs with tunable coordination environments through plasma-driven precursor decomposition and surface anchoring. Reproduced with permission from ref 56. Copyright 2021 Elsevier. (c) Plasma-activated construction of low-coordinated Cu SACs via plasma-induced defect formation and coordination restructuring. Reproduced with permission from ref 57. Copyright 2022 Royal Society of Chemistry.

Cold plasma also allows programmable tuning of single-atom coordination environments by generating reactive species that interact with the support and metal precursors. For instance, Li et al. developed a cold-plasma-enabled strategy for the synthesis of supported Pt SACs with tunable coordination environments.^56^ In this approach, high-energy electrons rapidly reduce Pt precursors into isolated Pt atoms at near-ambient temperatures, avoiding high-temperature annealing that commonly induces atom aggregation. Meanwhile, reactive species generated under different plasma atmospheres (N_2_, Ar, or air) introduce controllable heteroatom coordination, enabling the selective formation of Pt–N, Pt–O, or mixed Pt–N/O coordination structures (Figure 5b). By adjusting plasma parameters such as gas composition and treatment duration, the local coordination environment of Pt single atoms can be precisely controlled in a programmable manner. Electrochemical measurements demonstrate that coordination-engineered Pt single atoms exhibit markedly enhanced HER activity and stability compared with nanoparticle counterparts.

Beyond supported SACs, plasma-assisted coordination engineering can directly reconstruct metal sites in crystalline precursors. For instance, O_2_ plasma converts highly coordinated Cu centers in a Cu-based metal–organic framework (CuDBC) into isolated, low-coordinated Cu single atoms under mild conditions, offering an alternative to conventional high-temperature pyrolysis (Figure 5c).^57^ High-energy O_2_ plasma selectively disrupts Cu–O_4_ coordination, generating oxygen vacancies while preserving the overall framework. Simultaneously, plasma treatment induces surface roughening and hierarchical porosity without causing metal aggregation, thereby enhancing mass transport and reactant adsorption. The nonequilibrium nature of plasma enables precise tuning of metal coordination numbers (Cu–O_4_ → Cu–O_3_/Cu–O_2_) under mild conditions, eliminating the need for harsh thermal treatment or postetching steps.

This strategy has also been extended to construct unsaturated single-atom sites on monolayer transition-metal dichalcogenides.^58^ Nonequilibrium hydrogen plasma selectively dissociates metal-chalcogen bonds at low temperature (<80 °C), releasing metal atoms while preserving the structural integrity of the monolayer support. The highly reactive hydrogen species prevent atom aggregation and kinetically trap isolated metal atoms at well-defined surface positions, yielding high densities of coordinatively unsaturated single atoms without the need for high-temperature annealing or chemical precursors. Using monolayer MoS_2_ as a model, plasma treatment converts saturated fully coordinated Mo sites into isolated Mo single atoms with reduced coordination numbers (Mo–S_2_ or Mo–S_3_), while maintaining the crystalline basal plane. This approach has been further generalized to form W single atoms on WS_2_ monolayers under similar conditions.

These studies demonstrate that cold and nonequilibrium plasma technologies provide a unifying and versatile platform for precise coordination engineering of SACs across diverse material systems, from supported catalysts to crystalline metal–organic frameworks and 2D materials. Plasma-enabled reconstruction provides a powerful, low-temperature route to create highly active, coordinatively unsaturated metal centers that are otherwise difficult to access via conventional thermal or chemical methods.

#### 2.2.2 Plasma-Assisted Defect Engineering of SACs

Plasma-assisted defect engineering has emerged as a frontier strategy for catalyst design, enabling the controlled creation of metastable defects and anchoring sites under nonequilibrium conditions. Unlike conventional chemical or thermal treatments, plasma can selectively generate vacancies, edge sites, and functional groups that stabilize isolated metal atoms while preserving the underlying support structure, thereby enhancing atomic dispersion, electronic properties, and catalytic performance. For example, Chang and co-workers developed a flash CO_2_ plasma immersion strategy to simultaneously construct surface carboxyl defects and atomically dispersed Cu sites on graphitic carbon nitride (g-C_3_N_4_), demonstrating the unique capability of plasma to engineer defects beyond conventional chemical routes.^59^ Under nonequilibrium CO_2_ plasma irradiation, high-density energetic electrons and reactive CO_*x*_/O species selectively break C–N bonds in the g-C_3_N_4_ framework, generating abundant intrinsic carboxyl-type defect sites while preserving the polymer backbone (Figure 6a). These defects serve as robust anchoring sites for Cu atoms, preventing aggregation and enabling stable atomic dispersion without the structural damage typically induced by conventional post-treatments.

**Figure 6:**
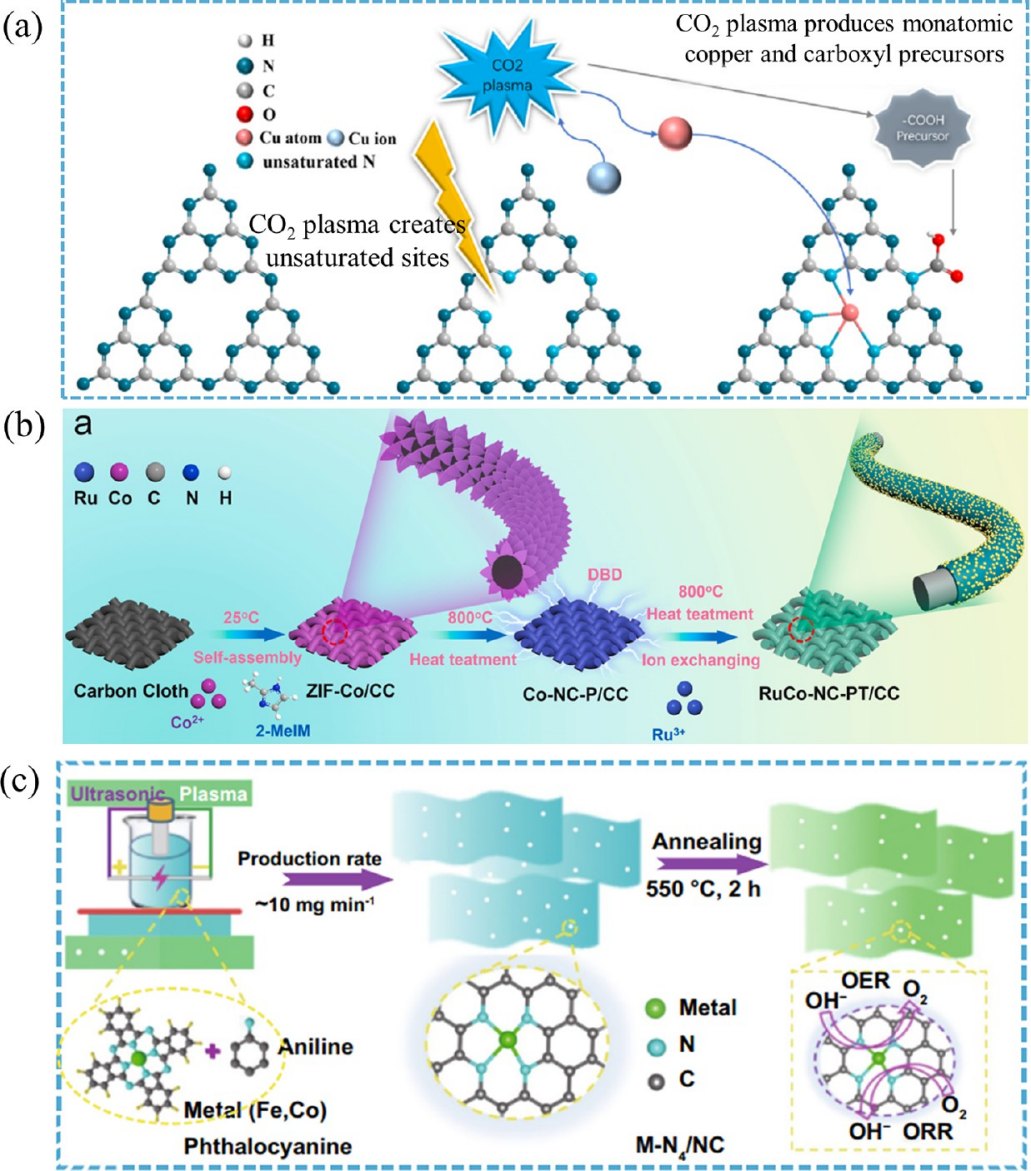
Plasma-assisted defect engineering: (a) Flash CO_2_ plasma immersion for the simultaneous construction of surface carboxyl defects and atomically dispersed Cu sites on graphitic carbon nitride. Reproduced with permission under a Creative Commons CC BY 4.0 from ref 59. Copyright 2022 Elsevier. (b) Plasma-assisted defect engineering for constructing a high-density Ru single-atom and RuCo alloy nanoparticle synergistic catalytic system. Reproduced with permission from ref 60. Copyright 2025 Elsevier. (c) Ultrasonication-assisted plasma engineering for the direct synthesis of single-atom M-N_4_/N-doped carbon catalysts. Reproduced with permission under a Creative Commons CC BY 4.0 from ref 61. Copyright 2021 Springer Nature.

Similarly, DBD plasma has been used to construct high-density Ru single atoms alongside RuCo alloy nanoparticles on defect-rich carbon supports.^60^ Plasma-generated vacancies, edge sites, and N-coordination motifs effectively immobilize Ru atoms, suppressing agglomeration and enabling high Ru loading (5.32 wt %) while simultaneously minimizing Ostwald ripening and nanoparticle agglomeration during subsequent thermal processing (Figure 6b). Tunable defect density further optimizes atomic utilization, electronic charge transfer, and hydrogen spillover, enabling exceptional HER performance at ampere-level current densities.

Ultrasonication-assisted plasma has also been applied to synthesize single-atom M–N_4_/N-doped carbon catalysts (M = Fe, Co), overcoming the complexity and high-temperature requirements of conventional pyrolysis methods.^61^ Under nonequilibrium plasma discharge, rapid carbonization of aniline generates a defect-rich nitrogen-doped carbon matrix with abundant edge sites and lattice imperfections, while preserving planar M–N_4_ coordination inherited from metal phthalocyanine precursors (Figure 6c). These plasma-induced carbon defects provide strong anchoring environments that kinetically stabilize isolated metal centers, preventing aggregation during both plasma processing and subsequent mild annealing.

Building on defect-driven SAC design, Xing et al. developed a solution plasma processing strategy to engineer defect and construct SACs with nearly quantitative metal utilization.^62^ In their study, high-energy electrons and reactive radicals simultaneously induce abundant oxygen vacancy defects (Ce^3+^/O_v_) on CeO_2_ nanosheets while reducing metal precursors into isolated Au single atoms, achieving a highly coupled process of defect formation and atomic trapping within minutes. The resulting oxygen vacancies act as robust anchoring sites, preventing aggregation and enhancing metal–support interactions, which also promote lattice oxygen activation and enable a photoenhanced Mars–van Krevelen pathway for low-temperature CO oxidation. Similarly, oxygen plasma functionalization has been applied to stabilize and activate Pt single atoms on CeO_2_ with high density and exceptional thermal stability.^63^ Oxygen plasma treatment induces pronounced surface nanostructuring and generates abundant oxygen-related defects, most notably surface peroxo (O_2_^2–^) species, which are absent on pristine or merely reduced CeO_2_ surfaces. These plasma-induced defective motifs act as strong anchoring centers that immobilize Pt atoms as isolated Pt^2+^ species, effectively suppressing surface diffusion and sintering even under high-temperature CO oxidation conditions. Compared with conventional thermally or ion-treated supports, plasma-functionalized CeO_2_ stabilizes single atoms more effectively through the combined effect of defect-rich structures and chemically active oxygen species.

### 2.3 Plasma-Driven Site Reconstruction

Under nonequilibrium plasma conditions, single-atom active sites can undergo dynamic structural evolution, involving continuous coordination rearrangement, ligand exchange and atomic migration. Such plasma-driven site reconstruction transforms initially coordinated metal centers into unsaturated and highly active configurations, modulating both local electronic structure and catalytic performance. Chen et al. demonstrated that cold plasma treatment can induce site reconstruction at the atomic scale, enabling the formation of Pt single atoms dynamically reconstructed on ultrafine Ru nanoclusters.^64^ During plasma exposure, high-energy electrons and reactive species simultaneously reduce metal precursors while suppressing thermally induced sintering, resulting in a nonequilibrium reconstruction in which Pt atoms preferentially migrate and anchor onto Ru nanocluster surfaces as isolated sites (Figure 7a). This plasma-driven site reconstruction generates Pt–Ru interfacial atomic configurations, which effectively modulate the local coordination environment and electronic structure of Ru. As a result, the reconstructed Pt single atoms act as atomic-scale modifiers, optimizing water adsorption and dissociation kinetics while preserving the high surface exposure of Ru nanoclusters. Such reconstruction processes may be dynamically reversible under reaction conditions, enabling regeneration of active configurations. Correspondingly, the catalyst shows negligible degradation after 8000 cyclic voltammetry (CV) cycles, and during 30 h operation at 20 mA cm^–2^, the overpotential increases from 17 to 55 mV, which can be largely restored after CV activation.

**Figure 7:**
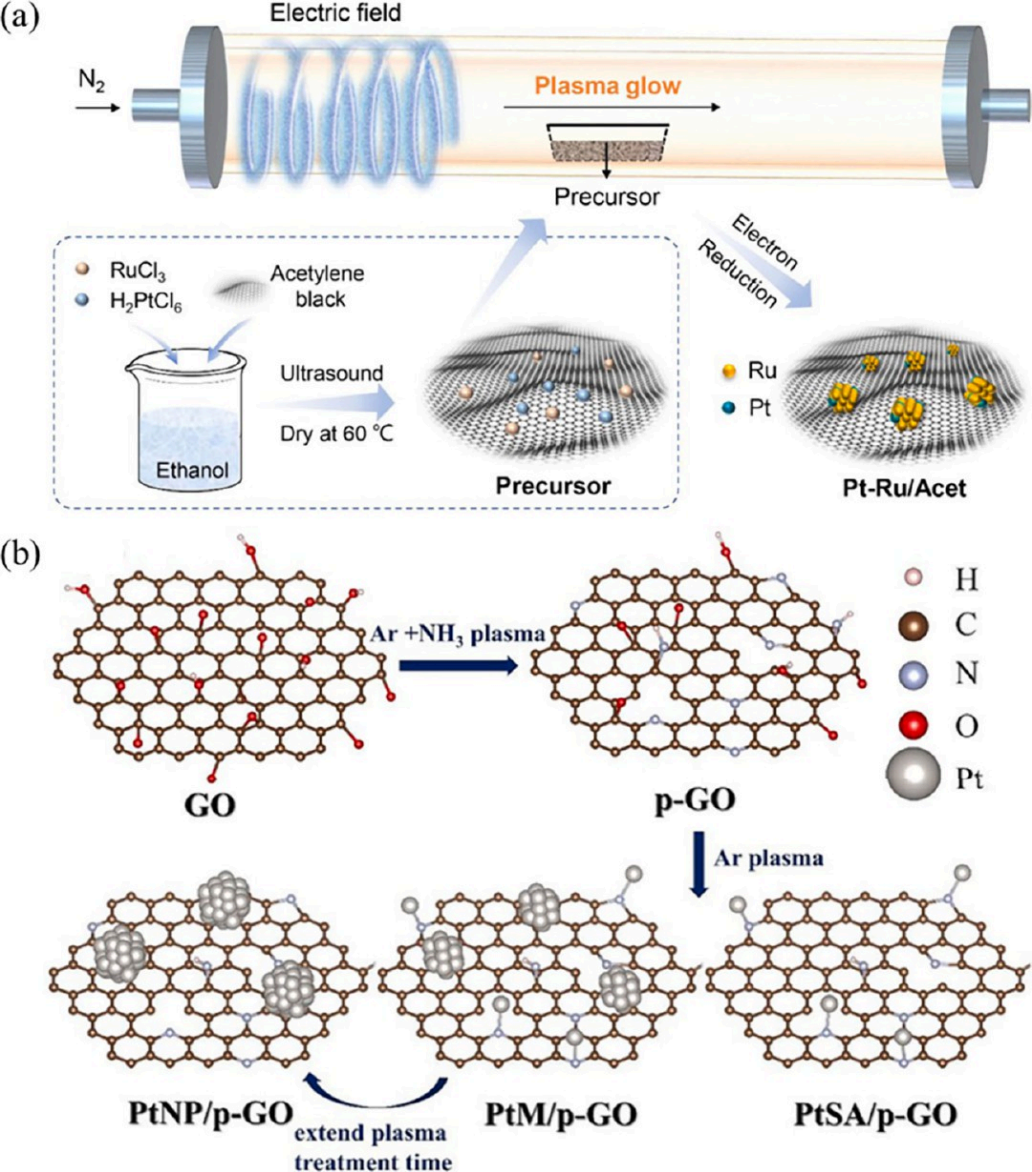
Plasma-driven site reconstruction: (a) Cold plasma-induced atomic-scale site reconstruction enabling the formation of dynamically reconstructed Pt single atoms on ultrafine Ru nanoclusters. Reproduced with permission from ref 64. Copyright 2022 Elsevier. (b) NTP-driven site reconstruction enabling the controlled coexistence and nuclearity evolution of Pt single atoms and NPs. Reproduced with permission from ref 65. Copyright 2023 Elsevier.

In addition, plasma-driven site reconstruction can regulate the nuclearity evolution of metal species in a controllable manner.^65^ Sequential plasma treatments first induce nitrogen doping and defect formation on graphene oxide, followed by plasma-driven reduction and restructuring of Pt precursors under nonequilibrium conditions (Figure 7b). By precisely tuning plasma parameters such as treatment time and precursor concentration, Pt species evolve from isolated single atoms to mixed single-atom/nanoparticle configurations, and ultimately to nanoparticles, revealing that plasma acts as an active regulator of metal nuclearity rather than a simple reduction tool, which dominates catalytic activity at low current densities. Importantly, plasma-induced defects and N-coordination sites guide the migration, anchoring, and reconstruction of Pt species, suppressing uncontrolled aggregation while allowing adaptive structural evolution. These dynamically reconstructed structures maintain a balance between isolated atoms and nanoparticle domains, contributing to stable catalytic performance over extended operation. As a result, the mixed Pt single- atom/nanoparticle catalyst exhibits excellent HER activity, with an overpotential of 18 mV at 10 mA cm^–2^ and 130 mV at 1000 mA cm^–2^, while maintaining stable operation at 1400 mA cm^–2^ for 24 h. Furthermore, the overpotential increases by only ∼3 mV after 2000 CV cycles, indicating that the dynamically reconstructed Pt species remain structurally stable during long-term operation.

Beyond nuclearity control, plasma-driven reconstruction can also modulate metal–support interfacial structures. Yang et al. demonstrated that hydrogen plasma pretreatment of Pt single-atom precursors supported on mechanically activated Al_2_O_3_ induces nonequilibrium restructuring of both Pt species and the alumina surface prior to conventional calcination and reduction.^66^ Plasma exposure partially reduces Pt^2+^ species, removes residual chloride ligands, and, critically, generates undercoordinated Al^3+^ sites and oxygen-related defects on the alumina support. These plasma-induced defects act as dynamic anchoring centers that guide the subsequent nucleation, migration, and reconstruction of Pt atoms into strongly bound, raft-like Pt nanostructures with enhanced metal–support interaction. Notably, hydrogen plasma uniquely promotes this reconstruction pathway, leading to a higher density of catalytically active interfacial Pt sites and improved stability under propane dehydrogenation conditions, compared with inert plasma treatments.

An important question in plasma-induced site reconstruction is whether these structural changes are reversible under reaction conditions. The dynamic plasma environment can induce migration and rearrangement of metal atoms, leading to transient reconstruction of active sites. In some cases, these processes are partially reversible, enabling regeneration of catalytically active configurations and mitigating irreversible sintering. However, excessive reconstruction or uncontrolled atom migration may still lead to gradual aggregation during prolonged operation. Therefore, understanding the reversibility and kinetics of plasma-induced structural evolution is crucial for the rational design of stable SACs in plasma-catalytic systems.

## 3 Plasma-Driven Reactions over SACs

Plasma provides an effective route for the activation of inert molecules with strong chemical bonds, while SACs offer well-defined active sites that interact with plasma-generated intermediates, thereby modulating reaction pathways and product formation (Figure 8). Unlike conventional thermal catalysis, plasma-driven systems operate under pronounced nonequilibrium conditions, generating energetic electrons, excited species and radicals that cannot be readily accessed by thermal activation alone. In this context, SACs serve as structurally uniform interaction centers that mediate the coupling between gas-phase plasma chemistry and surface reactions, enabling mechanistic insights into plasma–catalyst interactions and offering opportunities to tune reactivity through atomic-scale catalyst design.

**Figure 8:**
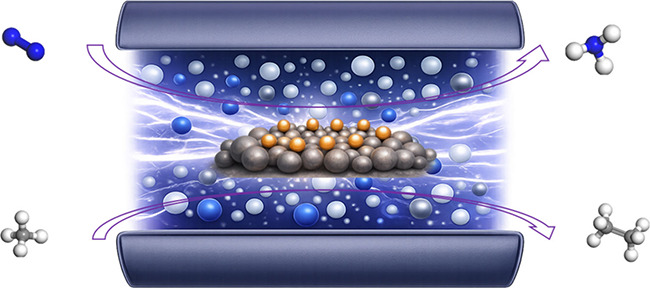
Schematic illustration of plasma-driven single-atom catalysis.

The stability of SACs under plasma reaction conditions is an important consideration. Although energetic electrons, ions, and radicals generated in plasma may induce atom migration or surface restructuring, strong metal–support interactions and well-defined coordination environments (e.g., M–N–C sites) effectively stabilize isolated metal atoms and suppress aggregation. Nevertheless, excessive plasma power or prolonged exposure may still promote atom diffusion or clustering, highlighting the importance of carefully controlling plasma parameters to balance catalytic activity and structural stability.

### 3.1 Plasma-Driven N_2_ Activation over SACs

The activation of N_2_ remains a fundamental challenge in catalysis due to the exceptional strength of the N≡N triple bond. NTP can generate vibrationally excited N_2_ molecules and reactive nitrogen radicals, significantly lowering activation barriers. SACs provide well-defined adsorption sites that can stabilize these activated nitrogen species, facilitating ammonia formation under mild conditions.

Wu et al. demonstrated a tandem plasma-electrocatalysis strategy that decouples nitrogen activation from ammonia synthesis, enabling efficient NH_3_ production directly from air and water at ambient conditions.^67^ In this system, a gliding arc plasma first converts inert N_2_ and O_2_ into reactive nitrogen oxide intermediates (primarily NO_2_^–^), thereby bypassing the high energy barrier associated with direct N_2_ activation (Figure 9a). These plasma-generated intermediates are subsequently reduced to NH_3_ over Co SACs with well-defined Co–N_4_ coordination, where isolated Co sites promote selective proton–electron transfer while suppressing the competing HER. As a result, the system achieves an NH_3_ yield rate of ∼1.43 mg_NH_3__ cm^–2^ h^–1^ with a Faradaic efficiency of ∼98% at −0.33 V vs RHE, and can reach ∼3.0 mg_NH_3__ cm^–2^ h^–1^ at −0.63 V vs RHE while maintaining stable operation for over 50 h. These values are significantly higher than those typically reported for conventional electrocatalytic N_2_ reduction systems, which often exhibit NH_3_ Faradaic efficiencies below ∼15%. By spatially and functionally separating plasma-driven molecular activation from electrocatalytic reduction, this strategy overcomes the intrinsic scaling limitations of conventional electrocatalytic nitrogen reduction.

**Figure 9:**
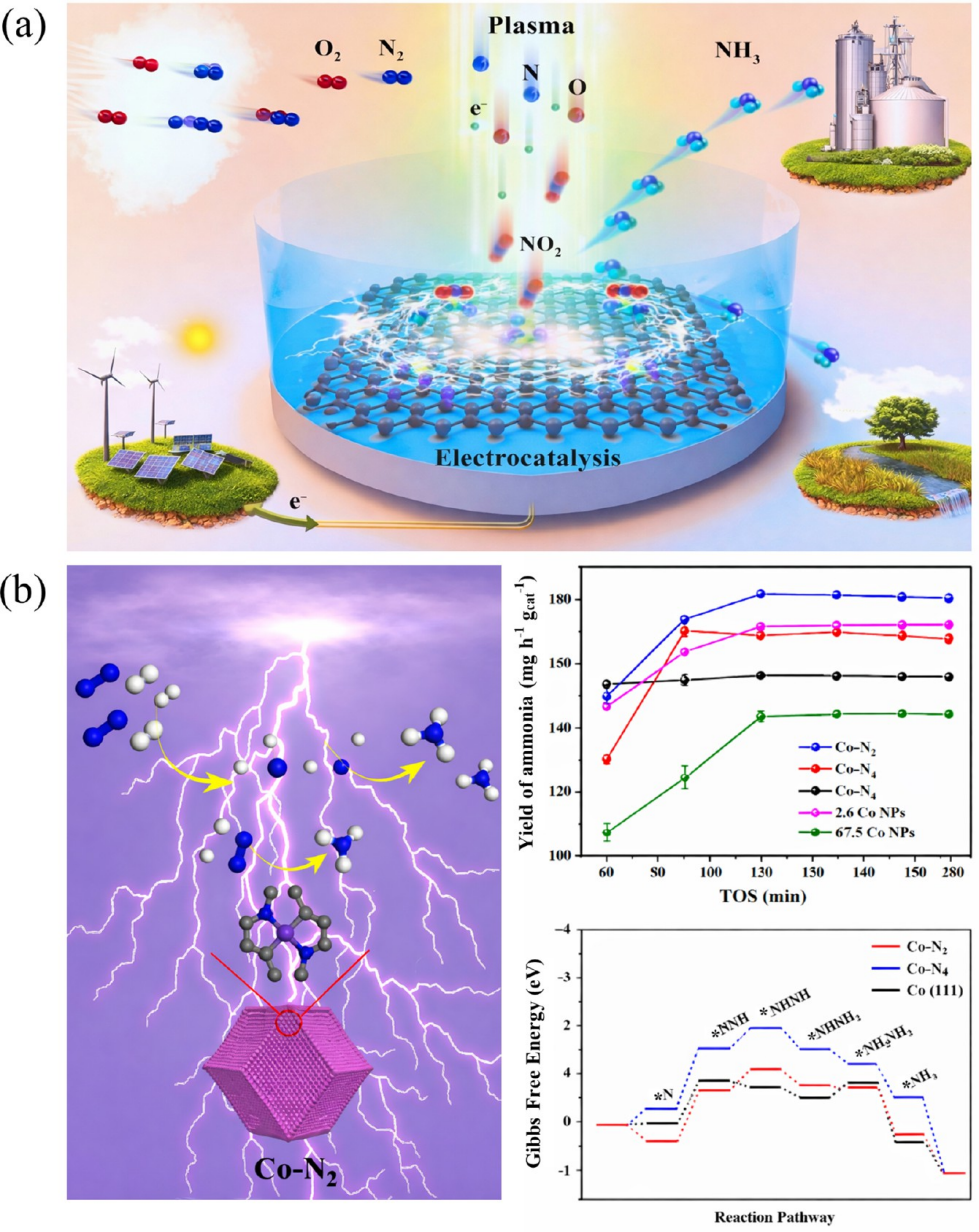
Plasma-assisted ammonia synthesis over SACs: (a) Tandem plasma-electrocatalysis strategy that decouples nitrogen activation and ammonia synthesis. Reproduced with permission from ref 67. Copyright 2021 Elsevier. (b) Synergistic effects of NTP and single-atom Co sites with different nitrogen coordination environments in plasma-assisted ammonia synthesis. Reproduced from ref 68. Copyright 2021 American Chemical Society.

NTP can also synergize directly with SACs to activate N_2_ without the formation of nitrogen oxide intermediates. In this context, the synergistic effects between NTP and Co-based catalysts, ranging from nanoparticles (NPs) to single-atom Co sites, have been systematically investigated for direct plasma-catalytic ammonia synthesis from N_2_ under mild conditions.^68^ By tuning the particle size of Co NPs and the nitrogen coordination environment of Co SACs (Co–N_4_, Co–N_3_, and Co–N_2_), this study demonstrates that catalyst nuclearity and coordination structure play a key role in governing plasma–catalyst interactions (Figure 9b). Notably, low-coordinated Co single atoms (Co–N_2_) exhibit the highest NH_3_ yield of 181 mg h^–1^g_cat_^–1^, outperforming Co–N_3_ (170 mg h^–1^ g_cat_^–1^) and Co–N_4_ (156 mg h^–1^g_cat_^–1^) SACs, as well as NPs such as 67.5 nm Co NPs (144 mg h^–1^ g_cat_^–1^). This enhancement is attributed to the more effective adsorption and dissociation of plasma-generated vibrationally excited N_2_ species on unsaturated Co–N_2_ sites, which lowers the activation barrier for N≡N bond cleavage. DFT calculations further corroborate these findings, confirming that reduced Co–N coordination facilitates N_2_ adsorption and dissociation in agreement with experimental observations. Moreover, the Co–N_3_ catalyst maintains stable ammonia production during plasma operation, indicating that the atomically dispersed Co sites remain structurally robust under NTP conditions. This work highlights that NTP can selectively couple with low-coordinated single-atom sites, establishing a structure-plasma synergy that enables efficient nitrogen activation beyond conventional catalytic pathways.

### 3.2 Plasma-Driven CH_4_ Activation over SACs

#### 3.2.1 Plasma-Driven CH_4_ Coupling to C_2+_ Hydrocarbons over SACs

Plasma-assisted CH_4_ activation benefits from radical-driven C–H bond cleavage. In such systems, SACs provide isolated and well-defined surface sites that can interact with plasma-generated CH_3_ and related intermediates, thereby influencing reaction pathways and reducing deep cracking and coke formation. The synergistic coupling between plasma-generated radicals and SACs enables methane conversion at low temperatures with enhanced selectivity. Liu et al. demonstrated that integrating DBD plasma with ceria-supported Pt SACs (Pt/CeO_2_-SACs) enables nonoxidative coupling of methane under near-ambient conditions.^69^ In this plasma-catalytic system, plasma serves as the primary driver for methane activation by generating vibrationally and electronically excited CH_4_ species at low temperatures (<150 °C), thereby bypassing the high activation barrier associated with C–H bond cleavage and facilitating the formation of CH_3_ radicals. The presence of atomically dispersed Pt sites further promotes the selective surface conversion of these plasma-activated intermediates into C_2_ hydrocarbons, while suppressing deep dehydrogenation and coke formation that commonly occur on Pt NPs (Figure 10a). Compared with plasma-only systems (CH_4_ conversion 7–37% and C_2_ selectivity 15–19%) and nanoparticulate Pt/CeO_2_ catalysts (CH_4_ conversion 7–40% and C_2_ selectivity 23–36%), the Pt/CeO_2_–SAC catalyst exhibits significantly improved performance, achieving a CH_4_ conversion of up to 43% with 46–54% C_2_ selectivity. Notably, the Pt/CeO_2_-SAC catalyst maintains stable activity during plasma operation, sustaining a CH_4_ conversion of ∼38–39% for 40 h on stream with only a slight decrease in C_2_ selectivity, indicating good resistance to deactivation under plasma conditions. This work highlights a clear plasma-single-atom synergy in which plasma-driven molecular activation and single-atom-controlled surface reactions cooperatively reshape methane conversion pathway, establishing PSAC as a promising strategy for low-temperature methane upgrading beyond the limitations of conventional thermal catalysis.

**Figure 10:**
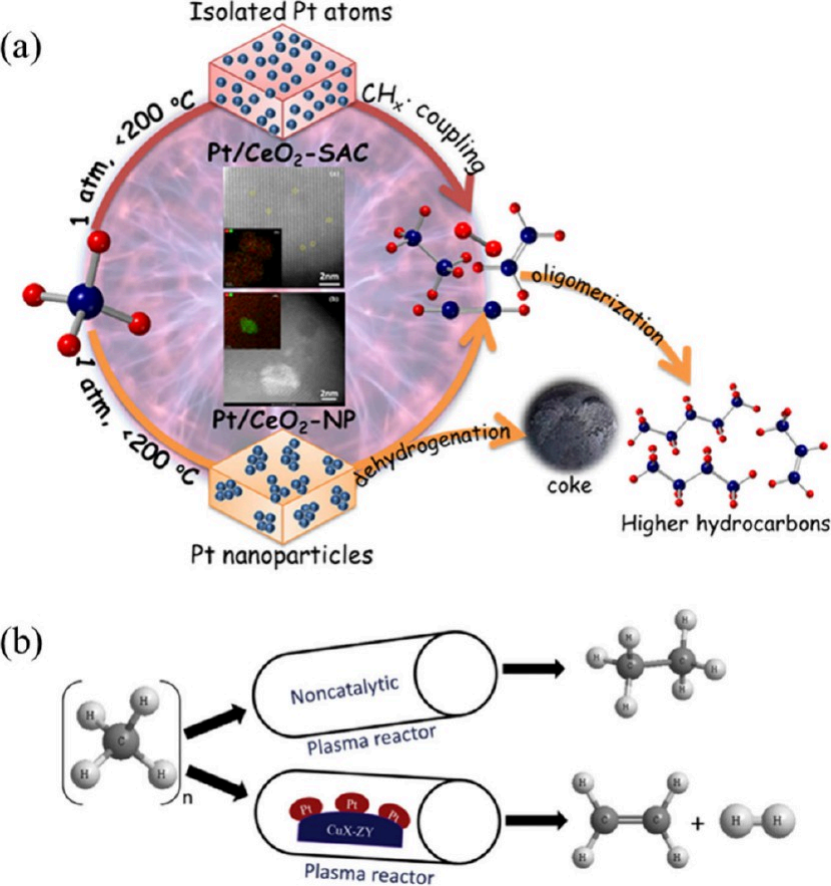
Plasma-driven methane conversion over Pt SACs: (a) Plasma-driven nonoxidative methane coupling over Pt SACs. Reproduced with permission from ref 69. Copyright 2022 American Chemical Society. (b) Plasma-enabled direct methane-to-ethylene conversion. Reproduced with permission from ref 70. Copyright 2022 Elsevier.

Building on this concept, further design strategies have been explored to improve selectivity toward specific C_2_ products, such as ethylene, by tuning catalyst composition and support interactions. A plasma-assisted single-atom catalysis strategy has been demonstrated for the direct nonoxidative conversion of methane to ethylene under mild conditions, addressing a longstanding challenge in methane activation and upgrading.^70^ By integrating DBD plasma with a rationally designed atomically dispersed Pt catalyst confined by CeO_2_ and a Cu-modified zeolite, methane activation is effectively decoupled from product selectivity control (Figure 10b). In this system, NTP serves as the primary driver for C–H bond activation through hydrogen abstraction, generating reactive CH_*x*_ radicals at low temperatures. The isolated Pt single atoms with a deliberately downshifted d-band center selectively promote C–C coupling pathways and facilitate rapid ethylene desorption, thereby suppressing overhydrogenation to ethane. Compared with plasma-only systems, which achieve ∼36.7% CH_4_ conversion but produce mainly ethane with negligible ethylene formation, the Pt SAC catalyst significantly enhances selectivity toward ethylene. Moreover, relative to nanoparticle-based catalysts (C_2_H_4_/C_2_H_6_ = ∼0.032), the Pt SAC delivers markedly improved performance, achieving ∼73.5% methane conversion and ∼23.9% ethylene yield with a high C_2_H_4_/C_2_H_6_ ratio exceeding 12. The catalyst also maintains stable performance during at least 12 h of continuous operation, indicating good structural robustness under plasma conditions. Spectroscopic characterization and electronic structure calculations further reveal that strong metal–support interactions and atomic dispersion play key roles in modulating radical–surface interactions. Overall, these results suggest that the combination of plasma-driven molecular activation and single-atom-controlled surface chemistry can overcome thermodynamic and kinetic constraints in methane upgrading, highlighting a promising strategy for C–H activation beyond the limitations of conventional thermal catalysis.

#### 3.2.2 Plasma Coupling of CH_4_ and H_2_O to Methanol over SACs

The direct coupling of methane and water to methanol offers an attractive and sustainable route for methane valorization, as it avoids external oxidants and enables the utilization of abundant natural gas resources. Methanol is a versatile platform chemical and energy carrier, yet conventional production relies on multistep, energy-intensive syngas processes. Activating the strong C–H bond in methane while selectively forming C–O bonds under mild conditions remains a significant challenge. Developing efficient CH_4_–H_2_O coupling strategies could enable low-carbon, decentralized methanol production and significantly advance green chemical manufacturing and carbon-neutral energy technologies.

Liu et al. demonstrated that coupling nonthermal CH_4_/H_2_O plasma with boron nitride (BN)-confined Fe SACs enables directional radical assembly for direct methane-to-methanol conversion under mild conditions.^71^ In this plasma-catalytic system, the plasma serves as the primary driver for generating abundant CH_3_ and OH radicals, while the atomically dispersed Fe sites act as adsorption and stabilization centers for these radicals. Defect-confined Fe single atoms embedded in BN preferentially capture OH radicals, forming Fe–OH motifs that subsequently promote rapid coupling with gas-phase CH_3_ via an Eley–Rideal pathway before radical recombination occurs (Figure 11). As a result, the plasma-Fe/BN system achieves a methanol production rate of ∼0.87 mmol h^–1^ with a liquid-phase selectivity of 91.6%, corresponding to an ∼2.5-fold enhancement compared with the plasma-only system (∼0.25 mmol h^–1^). In addition, the catalyst maintains stable methanol production for at least ∼6.5 h under continuous discharge conditions, indicating structural robustness of the atomically dispersed Fe sites. Experimental diagnostics combined with plasma kinetic modeling and DFT calculations reveal that the synergy between plasma-generated radicals and single-atom Fe sites enables controlled radical enrichment, stabilization, and directional assembly, resulting in both significantly enhanced methanol yield and selectivity compared with the plasma-only system. These findings highlight how plasma-driven molecular activation and single-atom sites can influence radical pathways, demonstrating the potential of plasma-assisted single-atom catalysis for selective C_1_ chemistry under mild conditions.

**Figure 11:**
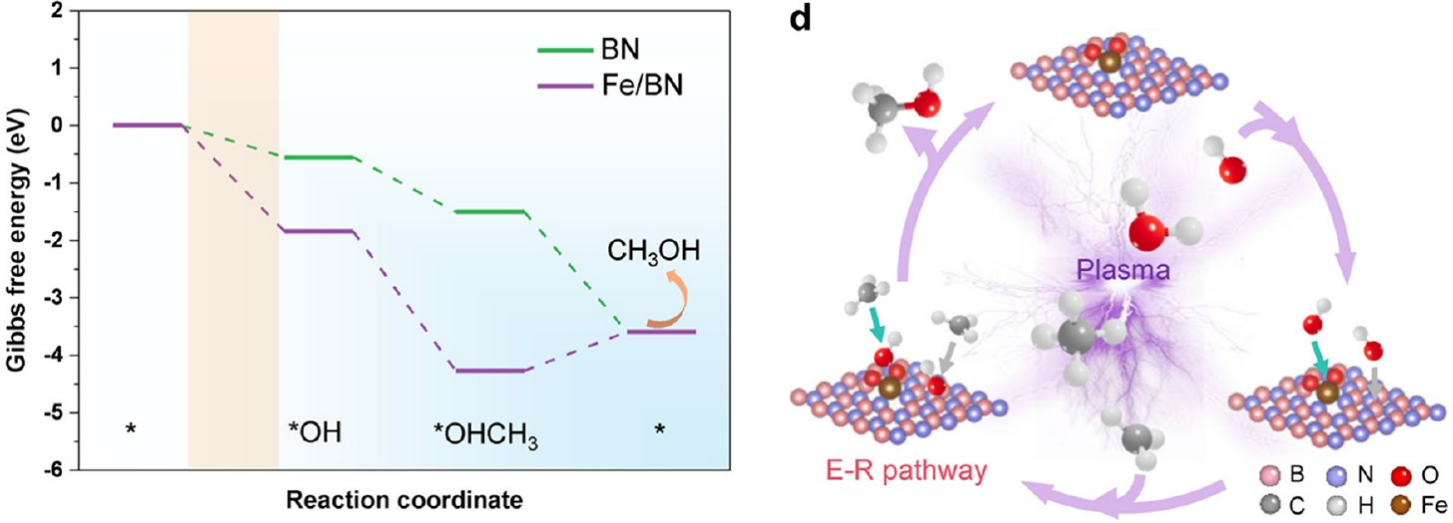
NTP conversion of CH_4_ and H_2_O into methanol over BN-confined Fe single atoms, enabling directional radical assembly. Reproduced with permission from ref 71. Copyright 2025 Wiley.

#### 3.2.3 Plasma-Driven Dry Reforming of CH_4_ and CO_2_ over SACs

Dry reforming of methane (DRM), which converts CH_4_ and CO_2_ into syngas (CO and H_2_), represents an attractive route for the simultaneous utilization of two major greenhouse gases. Compared with steam reforming of methane, DRM produces syngas with a low H_2_/CO ratio that is well suited for downstream Fischer–Tropsch synthesis and oxygenate production. However, this reaction is highly endothermic and typically requires high operating temperatures (>800 °C), leading to rapid catalyst deactivation caused by carbon deposition and metal sintering. Developing efficient DRM processes under milder conditions is therefore of great importance for sustainable syngas production and carbon-neutral energy conversion.

Diao et al. reported an atomically adjacent NiFe bimetallic catalyst that enables highly efficient and durable plasma-catalytic DRM at a substantially reduced temperature of 500 °C.^72^ By coupling DBD plasma with NiFe/MgAlO catalysts, the system demonstrates that plasma-induced nonequilibrium activation of CH_4_ and CO_2_ significantly lowers thermodynamic barriers while enhancing catalyst performance. Structural characterization reveals that Ni and Fe form adjacent atomic configurations, which synergistically enhance CO_2_ adsorption and surface oxygen availability. The resulting oxygen-rich environment accelerates the oxidation and removal of carbonaceous intermediates generated from plasma-enhanced CH_4_ dissociation, thereby suppressing coke accumulation, a major limitation in both thermal and plasma-assisted DRM. Under plasma-thermal coupling conditions, the NiFe/MgAlO catalyst achieves CH_4_ and CO_2_ conversions of 73.8% and 80.5%, respectively, outperforming the monometallic Ni/MgAlO catalyst under the same conditions (64.8% and 71.0%). In contrast, much lower conversions are obtained under thermal-only conditions at 500 °C (16.0% for CH_4_ and 22.2% for CO_2_) or plasma-only operation (38.1% and 23.4%, respectively), highlighting the strong synergistic effect between plasma activation and catalytic surface reactions. Furthermore, the NiFe catalyst maintains stable performance for over 100 h time-on-stream, demonstrating excellent resistance to deactivation. This work highlights the critical role of atomic-scale bimetallic site engineering in modulating plasma–surface interactions and carbon removal pathways, providing a rational strategy for the design of durable catalysts for plasma-driven C_1_ reforming chemistry.

### 3.3 Plasma-Driven Activation of Organic Molecules over SACs

The degradation and valorization of organic molecules, ranging from volatile organic compounds (VOCs) to polymeric plastic waste, represent central challenges for sustainable environmental and energy technologies. Conventional thermal catalytic approaches often require high temperatures and suffer from low selectivity and rapid catalyst deactivation. Plasma catalysis provides a nonequilibrium pathway to activate strong chemical bonds under mild conditions, yet plasma-only processes generally lack precise reaction control. Integrating NTP with SACs allows precise regulation of plasma-generated intermediates through well-defined atomic active sites, enabling efficient bond cleavage, selective transformation, and suppression of undesired side reactions. This approach offers a promising strategy for effective VOC oxidation and plastic recycling toward a circular and low-carbon economy.

Ye et al. demonstrated that coupling NTP with single-atom Ag_1_/MnO_2_ catalysts enables high-throughput and low-temperature oxidation of *n*-hexane, addressing key limitations of conventional VOC abatement technologies.^73^ In this plasma-catalytic system, NTP generates abundant reactive oxygen species and energetic electrons that initiate gas-phase fragmentation of *n*-hexane under mild conditions (∼120 °C), while atomically dispersed Ag sites anchored on MnO_2_ selectively adsorb and oxidize plasma-derived intermediates (Figure 12a). The incorporation of single Ag atoms induces lattice distortion and enriches surface oxygen vacancies, enhancing oxygen mobility and redox cycling between Mn^4+^ and Mn^3+^ states. As a result, the Ag_1_/MnO_2_ catalyst achieves a high *n*-hexane conversion of 96.3%, significantly higher than that obtained with plasma-only operation (77.2%), while thermal catalysis alone at the same temperature shows negligible activity. In addition, the system delivers a high energy yield of 74.1 g kWh^–1^ with minimal formation of ozone (<5 ppm) and NO_*x*_ (<20 ppm). Importantly, the catalyst exhibits remarkable operational stability, maintaining ∼96% conversion over 100 h of continuous plasma operation, indicating robust anchoring of Ag single atoms and stable plasma–catalyst interactions. In situ spectroscopic analyses and DFT calculations reveal that plasma-driven molecular activation and single-atom-controlled surface oxidation cooperatively lower reaction barriers and guide the reaction pathway toward complete oxidation. This work highlights the critical role of single-atom sites as selective sinks for plasma-generated intermediates, establishing plasma-assisted single-atom catalysis as a powerful strategy for efficient and environmentally benign VOC removal at low temperatures.

**Figure 12:**
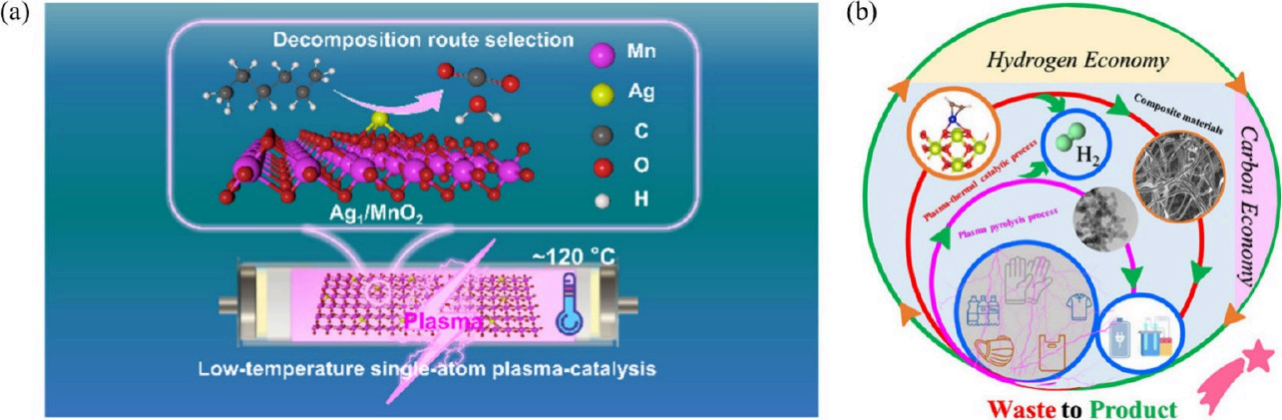
Plasma-driven activation of organic molecules: (a) NTP-assisted oxidation of *n*-hexane over single-atom Ag_1_/MnO_2_ catalysts. Reproduced with permission under a Creative Commons CC BY 4.0 from ref 73. Copyright 2025 American Chemical Society. (b) Plasma-assisted plastic waste upcycling into hydrogen and high-value carbon nanomaterials. Reproduced with permission under a Creative Commons CC BY 4.0 from ref 74. Copyright 2024 Wiley.

Beyond VOC oxidation, plasma catalysis also demonstrates highly promise for the activation and degradation of large and complex molecules such as plastics. A plasma-enabled strategy has been developed for sustainable plastic waste upcycling, demonstrating that NTP can rapidly decompose diverse and mixed plastic wastes into hydrogen-rich gases and high-value carbon nanomaterials (Figure 12b).^74^ In the catalyst-free plasma process, highly energetic electrons and reactive species directly cleave polymer C–C and C–H bonds, achieving H_2_ yields and selectivities far exceeding those of conventional thermal pyrolysis. More importantly, coupling plasma pyrolysis with a downstream thermal catalytic stage incorporating atomically dispersed M/CeO_2_ (M = Fe, Co, Ni) SACs further enhances hydrogen production while maximizing metal utilization efficiency. The CeO_2_ support provides defect-rich anchoring sites that stabilize isolated metal atoms, enabling efficient dehydrogenation of plasma-generated hydrocarbon intermediates and suppressing catalyst deactivation. As a result, the integrated plasma-thermal catalytic system achieves H_2_ yields of up to 46.7 mmol g_plastic_^–1^ (∼64.4% of the theoretical yield), while maintaining stable performance over 10 successive reaction cycles. Notably, a 1 wt % Fe/CeO_2_ SACs delivers H_2_ yields comparable to those of nanoparticle-based catalysts with 10-fold higher metal loading, highlighting the unique synergy between plasma activation and single-atom catalysis. This work establishes plasma-SAC integration as a rapid, highly promising, effective route for scalable chemical recycling of plastics, toward a circular hydrogen and carbon economy.

## 4 Challenges and Future Perspectives in Plasma-Driven Single-Atom Catalysis

PSAC has emerged as a powerful and versatile paradigm that transcends the limitations of conventional thermal catalysis. Owing to its nonequilibrium nature, plasma enables the rapid activation of inert molecules with strong chemical bonds under mild conditions, while also offering unique capabilities for SACs construction, defect engineering, coordination modulation, and dynamic site reconstruction. When coupled with atomically precise catalytic sites, plasma–catalyst interactions can be rationally disentangled and harnessed to steer reaction pathways with enhanced activity and selectivity. Importantly, the synergy between plasma chemistry and SAC creates opportunities to access metastable active sites, regulate radical-mediated processes, and design structurally explicit catalytic systems. With continued advances in catalyst design, in situ characterization, and data-driven approaches, PSAC is poised to play a transformative role in energy conversion and sustainable chemical manufacturing.

Despite these advances, PSAC remains at an early stage, facing several fundamental challenges. Key limitations include an incomplete mechanistic understanding of plasma–catalyst interactions, insufficient in situ and operando characterization techniques, and the lack of standardized metrics for evaluating plasma-driven activity and energy efficiency. Addressing these issues requires systematic efforts to establish unified descriptors, benchmarking protocols, and structure-performance relationships tailored to nonequilibrium plasma systems.

Beyond current demonstrations in ammonia synthesis, methane activation, and plastic recycling, PSAC holds considerable promise for a broader range of chemical transformations. Reactions that are thermodynamically constrained under conventional conditions, such as CO_2_ hydrogenation to methanol or higher alcohols, and selective C–C coupling may benefit from plasma-induced activation pathways. The ability of plasma to generate highly reactive intermediates provides opportunities to bypass kinetic and thermodynamic limitations, thereby opening reaction routes inaccessible in traditional catalysis.

Expanding the library of SACs for plasma catalysis, together with precise control over their coordination environments, electronic structures, and metal–support interactions, will be essential for unlocking new reaction pathways and enhancing product selectivity. Particularly, engineering defect structures and interfacial interactions can create tunable active sites that selectively stabilize key intermediates under plasma conditions.

Advancing PSAC also critically depends on the development of advanced in situ and operando characterization techniques capable of probing dynamic processes under realistic plasma environments. Integrating plasma reactors with synchrotron-based X-ray absorption spectroscopy, ambient-pressure X-ray photoelectron spectroscopy, and high-resolution electron microscopy enables direct observation of transient species, evolving coordination environments, and genuine active sites. In particularly, plasma-compatible in situ/operando spectroscopy that simultaneously monitors plasma-phase intermediates and catalyst surface structures will be essential for elucidating the dynamic interplay between plasma-generated species and atomically dispersed metal sites, providing critical insights for the rational design of robust and efficient PSAC systems.

Furthermore, given that plasma inherently generates a wide spectrum of reactive species, future efforts should focus on multisite-enabled catalytic systems that can selectively interact with distinct plasma-derived intermediates. By engineering catalysts with multiple atomic sites or synergistic active centers, it becomes possible to spatially and chemically decouple key elementary steps, enabling selective adsorption, activation, and coupling of intermediates. This multisite strategy provides a powerful framework for steering complex reaction networks toward desired products, thereby enhancing activity, selectivity, and energy efficiency.

Beyond experimental advances, a critical bottleneck in PSAC lies in the limited realism of current theoretical models. Most simulations rely on ground-state approximations and idealized environments, failing to capture the intrinsic nonequilibrium nature of plasma systems. To address this limitation, future efforts should explicitly incorporate plasma-specific effects into catalytic modeling, including excited-state species, electron-induced surface processes, and dynamic interactions between plasma-generated species and active sites. Advanced computational frameworks that couple plasma kinetics with surface chemistry, such as multiscale and nonequilibrium simulations, are essential to accurately describe these complex systems. Elucidating how excited species interact with atomically dispersed metal sites at the electronic level will provide critical insights into plasma–catalyst synergy. These developments are also crucial for enabling data-driven and AI-assisted catalyst design.

In conclusion, PSAC represents a forward-looking paradigm in which nonequilibrium plasma chemistry and atomic-level catalysis converge, offering new opportunities to reshape catalyst design and energy conversion strategies beyond the limits of traditional catalysis.
